# Molecular characterization and protective efficacy of silent information regulator 2A from *Eimeria tenella*

**DOI:** 10.1186/s13071-016-1871-0

**Published:** 2016-11-25

**Authors:** Hui Dong, Sihan Yang, Qiping Zhao, Hongyu Han, Shunhai Zhu, Xuelong Zhu, Cong Li, Ziwen Wang, Weili Xia, Qifei Men, Liangyu Yang, Bing Huang

**Affiliations:** 1 Shanghai Veterinary Research Institute, Chinese Academy of Agricultural Science, Key Laboratory of Animal Parasitology of Ministry of Agriculture, Shanghai, 200241 China; 2 College of Animal Science and Technology, Yunnan Agricultural University, Kunming, 650201 China; 3 Jiangsu Co-Innovation Center for Prevention and Control of Important Animal Infectious Diseases and Zoonoses, Yangzhou, 225009 China

**Keywords:** Coccidia, *Eimeria*, Silent information regulator 2, Recombinant plasmid

## Abstract

**Background:**

Silent information regulator 2 (SIR2) proteins are a family of NAD + -dependent protein deacetylases that are considered potential targets for anti-parasitic agents. In this study, we cloned and characterized SIR2A of the protozoan parasite *Eimeria tenella* (EtSIR2A) and investigated its protective efficacy as a DNA vaccine.

**Methods:**

The EtSIR2A gene encoding 33.37 kDa protein from *E. tenella* second-generation merozoites was cloned, and recombinant EtSIR2A protein (rEtSIR2A) was produced in an *Escherichia coli* expression system. The rEtSIR2A was used to immunize rabbits. Anti-rEtSIR2A antibodies were used to determine the immunolocolization of EtSIR2A in the parasite by immunofluorescence assay (IFA). Transcript and protein expression of EtSIR2A in different development stages of *E. tenella* were observed by quantitative real-time PCR (qPCR) and western blot (WB) analysis, respectively. The recombinant plasmid pCAGGS-EtSIR2A was constructed and its efficacy against *E. tenella* infection in chickens was evaluated.

**Results:**

qPCR and WB analysis revealed EtSIR2A expression was developmentally regulated at both the mRNA and protein levels. EtSIR2A mRNA levels were higher in unsporulated oocysts than at other developmental stages, including sporulated oocysts, sporozoites and second-generation merozoites. In contrast, EtSIR2A protein expression levels were highest in second-generation merozoites, moderate in unsporulated oocysts and sporulated oocysts and lowest in sporozoites. Immunostaining with anti-rEtSIR2A antibody indicated that EtSIR2A was mainly located in the cytoplasm of sporozoites and second-generation merozoites, and was strongly expressed during first stage schizogony. Animal-challenge experiments demonstrated that immunization with pCAGGS-EtSIR2A significantly increased average body-weight gain, and decreased mean lesion score and oocyst output in chickens.

**Conclusions:**

These results suggest that EtSIR2A may play an important role in parasite cell survival and may be an effective candidate for the development of new vaccines against *E. tenella* infection in chickens.

**Electronic supplementary material:**

The online version of this article (doi:10.1186/s13071-016-1871-0) contains supplementary material, which is available to authorized users.

## Background

Avian coccidiosis is an intestinal disease caused by infection with any of several species of the protozoan genus *Eimeria,* and represents an economically important parasitic infection for the poultry industry worldwide [1]. The main methods for controlling coccidiosis in recent decades have been prophylactic chemotherapy, using ionophores and synthetic drugs [2]. However, the development of resistance to anti-coccidial drugs and increasing public pressure to limit the use of chemicals in animal feed continues to drive the development of anti-coccidial vaccines [3], including live vaccines. However, there are disadvantages to live vaccines including environmental contamination, high production expenses and an atavistic possibility of coccidiosis [4, 5]. These drawbacks have driven the development of new control strategies.

Silent information regulator 2 (SIR2) enzymes, or sirtuins, comprise a family of NAD + -dependent deacetylases that are evolutionarily conserved in all phyla, from bacteria to higher eukaryotes [6, 7]. In the past few years, sirtuins have been shown to be involved in numerous biological processes, including heterochromatin formation, gene silencing, DNA repair, development, longevity, metabolism, adipogenesis and apoptosis [8, 9]. SIR2 has already been identified in various parasites, including apicomplexans (*Plasmodium*, *Toxoplasma*, *Cryptosporidium*), kinetoplastids (*Leishmania*, *Trypanosoma*), and an amoebozoan (*Entamoeb*a) [10]. Previous results showed that SIR2 promoted parasite survival under various conditions [11–16], and it has thus emerged as a novel anti-parasitic therapeutic target [10, 17]. Most apicomplexans possess two sirtuins, SIR2A and SIR2B, both of which are included in the *Eimeria tenella* (Et) genome database (GeneDB) [18]. The SIR2A gene of *E. tenella* (EtSIR2A) was first identified by Yan et al. [19], but its role in *E. tenella* and its regulation during the life-cycle of the parasite remains poorly known. In the present study, we cloned and characterized EtSIR2A and investigated its protective efficacy as a DNA vaccine.

## Methods

### Parasites, cells, plasmids, and animals

The Shanghai strain of *E. tenella* was isolated from a sample collected on a chicken farm in Shanghai, China, in the 1980s and subsequently maintained in our laboratory [20]. Parasites were propagated by passage through coccidia-free 2-week-old chickens, as described previously [21]. Unsporulated and sporulated oocysts were obtained and purified using standard procedures [22, 23]. Sporozoites were prepared from cleaned sporulated oocysts by in vitro excystation, and purified by chromatography over columns packed with nylon wool and DE-52 cellulose [24]. Second-generation merozoites were collected and purified from the caecal mucosa of chickens at 112 h post-inoculation (p.i.) with 1 × 10^5^ sporulated oocysts per bird [22].

The chicken embryo fibroblast cell line DF-1 was cultured in Dulbecco’s modified Eagle’s medium (DMEM) (Invitrogen, Carlsbad, USA) supplemented with 10% fetal bovine serum (FBS). The eukaryotic expression vector pCAGGS was kindly provided by Dr. G.Z. Tong (Shanghai Veterinary Research Institute, Shanghai, China).

Yellow feathered broilers at 1 day old were kept in wire cages under coccidia-free conditions and provided with coccidiostat-free feed and water *ad libitum*. The chickens were moved to an animal-containment facility prior to challenge with virulent oocysts.

### Cloning and sequence analysis of EtSIR2A

The open reading frame of EtSIR2A was amplified by polymerase chain reaction (PCR) from cDNA of *E. tenella* second-generation merozoites using a pair of primers designed based on the sequence obtained from *E. tenella* GeneDB (http://www.genedb.org/Homepage/Etenella) (ID: ETH 00033350).

The specific PCR primers were: forward primer, 5′-GCG AAT TCA TGG GCC AGT GGT TAA CAT-3′; reverse primer, 5′-GCC TCG AGT CAT TCA TTT TCC CCT GGG-3′, containing *EcoR*I and *Xho*I restriction sites (underlined), respectively. The PCR mixture (50 μl) contained 25 μl of 2× *Taq* PCR Master Mix (Tiangen Biotech, Beijing, China), 2 μl of cDNA template, 2 μl of forward and reverse primers (10 μM) each, and deionized water up to 50 μl. The amplification conditions were 95 °C for 3 min; 35 cycles of 95 °C for 30 s, 50 °C for 30 s, 72 °C for 1 min, and 10 min at 72 °C. The PCR products were gel purified (Tiangen) and subcloned into the PMD18-T vector (TaKaRa, Dalian, China). TIANprep Mini Plasmid Kit (Tiangen) preparations of the recombinant plasmid were analyzed by gel electrophoresis. Positive recombinant clones were subjected to DNA sequencing by Invitrogen (Shanghai, China).

Analyses of the cDNA and deduced amino acid sequences of EtSIR2A were carried out as described previously [25]. Briefly, the full-length cDNA sequence of EtSIR2A gene was analyzed for similarity using the BLAST programs at the National Center for Biotechnology Information (http://www.ncbi.nlm.nih.gov/BLAST/) and the genome sequence of *E. tenella* (http://www.genedb.org/Homepage/Etenella). The deduced amino-acid sequence and molecular mass were obtained using translate tool software at the ExPASy server of the Swiss Institute of Bioinformatics (http://www.expasy.org/tools/protparam.html). The signal peptide, transmembrane (TM) regions and protein motifs were predicted using SignalP (http://www.cbs.dtu.dk/services/SignalP/), TMHMM (http://www.cbs.dtu.dk/services/TMHMM-2.0/), and Motifscan (http://hits.isb-sib.ch/cgi-bin/motif_scan), respectively. Multiple sequence alignment was performed used the program Clustal W (http://www.ebi.ac.uk/Tools/msa/clustalw2/).

### Expression and purification of recombinant EtSIR2A

The PCR product of EtSIR2A was digested with the restriction endonucleases *EcoR*I and *Xho*I, and cloned into the prokaryotic expression vector pET-28a (+) (Novagen, Merck KGaA, Darmstadt, Germany). The recombinant plasmid was confirmed by DNA sequencing and transformed into *Escherichia coli* BL21 (DE3) (Promega, Madison, USA). Bacteria with the recombinant pET-28a (+) plasmid were induced with 1 mM isopropyl-β-D-thiogalactopyranoside (IPTG; Sigma, Louis, USA) at 37 °C for 6 h, collected by centrifugation at 10,000× *g* for 15 min, and sonicated on ice. The supernatant was collected, and recombinant protein was purified using a His Bind Purification kit (Novagen). Purified protein lysate was analyzed by 12% sodium dodecyl sulfate–polyacrylamide gel electrophoresis (SDS-PAGE) and its concentration was determined using a BCA protein assay kit (Novagen). The purified protein was stored in aliquots at -20 °C until further use.

### Production of polyclonal sera against recombinant EtSIR2A

Two-month-old rabbits were immunized subcutaneously with 0.2 mg of purified rEtSIR2A emulsified in an equal volume of Freund’s complete adjuvant (Sigma), followed by three booster injections with proteins emulsified in equal volumes of Freund’s incomplete adjuvant at 2-week intervals. Serum was separated from the rabbit blood 7 days after the final immunization. Serum collected before protein injection was used as negative control serum. The anti-sera were stored at -80 °C for subsequent use.

### EtSIR2A transcript levels in different developmental stages of *E. tenella*

Total RNA was extracted from parasites in four stages (unsporulated oocysts, sporulated oocysts, sporozoites, and second-generation merozoites) using TRIzol (Invitrogen) and treated with DNase I (Invitrogen) to exclude interference from DNA. The RNA was quantified by spectrophotometry with a BioPhotometer (Eppendorf, Hamburg, Germany) and its integrity was verified by agarose gel electrophoresis. Two micrograms of total RNA was reverse transcribed to cDNA using SuperScript II reverse transcriptase (Invitrogen) and random primers. Quantitative real-time PCR (qPCR) was performed with SYBR-Green I fluorescence (TaKaRa) using a Bio-Rad iQ5 instrument (BioRad, Hercules, USA). The PCR primers for EtSIR2A were as follows: 5′-AAA AGA AAC TTC CTC CCA-3′and 5′-AAT CCT GTC TCC TCC AAA-3′. Three housekeeping genes of *E. tenella*, 18S rDNA, *β-actin* and *gapdh*, were used as reference genes for the purposes of normalization [26–28]. The qPCR reaction mixture (20 μl) contained 10 μl SYBR® Premix Ex Taq™ (2×) (TaKaRa), 1 μl (0.2 μM) of each primer, 1 μl of cDNA template and 7 μl RNase-free distilled H_2_O. Reactions were conducted under the following conditions: 95 °C for 15 s, 40 cycles of 95 °C for 5 s, 60 °C for 10 s, and 72 °C for 15 s. All assays were done in triplicate and the relative expression of EtSIR2A in different developmental stages was calculated using the 2^-ΔΔCt^ method [29].

### EtSIR2A protein expression in four development stages of *E. tenella*

Total protein was prepared from unsporulated oocysts, sporulated oocysts, sporozoites and second-generation merozoites using cell-lysis buffer for western blotting and immunoprecipitation (IP) (Beyotime, Haimen, China). Protein concentrations were determined using a BCA Protein Assay Kit (Beyotime). Thirty micrograms of each sample were subjected to SDS-PAGE on a 12% gel and then transferred to polyvinylidene difluoride transfer membranes (Millipore, Bedford, USA) at 0.28 A for 2.5 h at 4 °C. Membranes were blocked with 5% (w/v) skimmed milk powder in phosphate-buffered saline (PBS) overnight at 4 °C, rinsed three times for 10 min with PBS containing 0.1% Tween 20 (PBST), and incubated in either rabbit polyclonal anti-rEtSIR2A (1:100) or mouse monoclonal anti-tubulin (Beyotime, 1:2,000) diluted in PBS for 2 h at room temperature. After three washes in PBST, membranes were probed with IRDye 800CW goat anti-rabbit IgG (LI-COR Biosciences, Lincoln, USA, 1:10,000) or IRDy 680RD donkey anti-mouse IgG (LI-COR Biosciences, 1:10,000) diluted in PBS for 1 h at room temperature in a dark chamber. After a further three washes in PBST, the membranes were washed three times in PBS and scanned using an Odyssey® Infrared Imaging System (LI-COR Biosciences). For comparative quantitative protein expression profile analysis, the resulting images were analyzed by the software program as specified by Odyssey. Tubulin was used as an internal reference for protein extracts at each stage.

### Immunolocalization of EtSIR2A in parasites by indirect immunofluorescence assay

EtSIR2A protein localization was performed by immunofluorescence assay (IFA), as described previously [25], with slight modification. Briefly, DF-1 cells (3 × 10^5^ cell per well) were seeded in six-well plates (Corning, Corning, USA) with pre-coated sterile coverslips and cultured in complete medium (CM, DMEM containing 10% FBS, 100 U/ml penicillin/streptomycin, 2 mM L-glutamine) at 37 °C, 5% CO_2_ for 24 h. Freshly excysted sporozoites were incubated for 1 h at 41 °C in CM or PBS. Sporozoites in CM were added to adherent cells at a ratio of one sporozoite per cell. Infected DF-1 cells were collected at different times (12–72 h) p.i. for fixation. Sporozoites in PBS or infected DF-1 cells were washed with PBS for 10 min, fixed in 2% paraformaldehyde for 20 min, permeabilized with 1% Triton X-100 in PBS for 15 min, and then blocked with 2% bovine serum albumin in PBS overnight at 4 °C. The coverslips were then incubated with rabbit anti-rEtSIR2A antibody at a dilution of 1:100 for 1 h at 37 °C, and further incubated for 1 h with goat anti-rabbit IgG fluorescein isothiocyanate-conjugated antibody (1:500 dilution; Sigma) in a moist, dark chamber. Cell nuclei were labeled with 10 μg/ml 4,6-diamidino-2-phenylindole (DAPI, Beyotime) for 10 min. The coverslips were washed three times in PBST after every step. The coverslips were finally mounted on glass slides using 60 μl of Fluoromount Aqueous Mounting Medium (Sigma) and observed under a fluorescence microscope (Nikon, Tokyo, Japan). Second-generation merozoites purified from the ceca of chickens were incubated for 1 h at 41 °C in CM and were prepared for immunofluorescence observation using the same way as described above.

### Construction of pCAGGS-EtSIR2A DNA vaccine and its expression in vitro

The EtSIR2A PCR products were cloned into the pCAGGS vector and designated as pCAGGS-EtSIR2A. The constructed plasmid was identified to have the correct orientation by sequencing and was purified using a Qiagen Plasmid Giga Kit (Qiagen Biotech, Beijing, China). The plasmid concentration was determined by spectrophotometry at 260 nm.

A monolayer of 80–90% confluent DF-1 cells in six-well plates was transfected with 4 μg of pCAGGS-EtSIR2A or pCAGGS using Lipofectamine 2000 (Invitrogen). Briefly, DNA and the transfection reagent were mixed (10 ml lipofectamine 2000 and 4 μg DNA), incubated at room temperature for 30 min, and added to the cells. Six hours later, the DNA-transfection reagent mixture was replaced by DMEM containing 10% FBS. At 48 h post-transfection, the expression of encoded EtSIR2A protein from these plasmids was confirmed by indirect IFA and western blotting of transfected DF-1 cells.

### DNA vaccination and challenge infection in chickens

One hundred 1-week-old chickens were randomly divided into four groups of 25 chickens each. Groups 1 and 2 were immunized with 100 μg of pCAGGS-EtSIR2A and pCAGGS diluted in TE buffer (10 mM Tris-HCl pH 7.6, 1 mM EDTA), respectively. Groups 3 and 4 were injected with sterile TE buffer at the same injection site and were used as a challenged control (positive control) and unchallenged control group (negative control), respectively. All the groups were inoculated twice at 1-week intervals by intramuscular injection in the leg. At 21 days old, all chickens except the unchallenged control group were inoculated orally with 1 × 10^4^ sporulated oocysts of *E. tenella* Shanghai strain. All chickens were slaughtered at day 8 post-challenge to evaluate the lesion score, as described previously [30]. Chicken body weights were measured at days 0 and 8 post-challenge. Faeces from each group were collected separately at days 6–8 post-challenge. Oocyst shedding per bird was determined using a McMaster chamber [31, 32]. Each faecal sample was counted three times using the same methods. Oocyst decrease ratio (%) was calculated as follows: (the average number of oocysts per bird from challenged control group-the average number of oocysts per bird from vaccinated group)/the average number of oocysts per bird from challenged control group × 100.

### Statistical analysis

All data were presented as the mean ± standard deviation (SD). The significance of differences among the groups was evaluated by one-way ANOVA with SPSS 13.0 software (SPSS Inc., Chicago, IL, USA). The Duncan’s multiple range test was used to analyze differences between the mean values and a value of *P* < 0.05 was considered significant.

## Results

### Cloning and sequence analysis of EtSIR2A

The EtSIR2A gene was isolated by PCR using cDNA from second-generation merozoites from the *E. tenella* Shanghai strain. After cloning and sequencing, a predicted 909-bp product was obtained and analyzed by BLASTn. The sequence displayed 100% identity with the known NAD^+^-dependent deacetylase (SIR2) gene from the *E. tenella* Houghton strain (GenBank: XM_013377851), suggesting that the EtSIR2A gene from the *E. tenella* Shanghai strain had been successfully amplified (GenBank: KU871068).

**Fig. 1:**
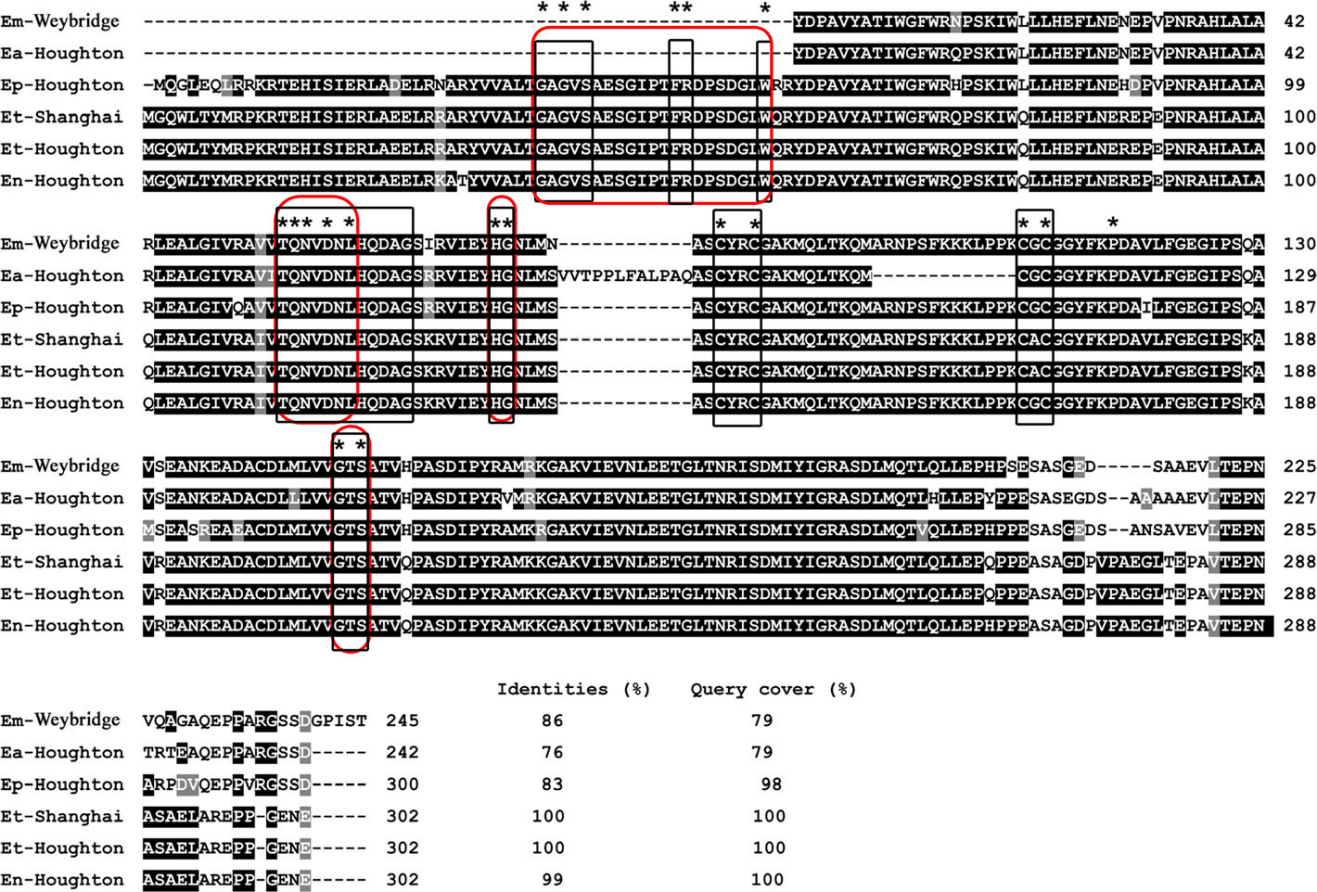
Alignment of silent information regulator 2 (SIR2) amino acid sequences from five *Eimeria* species. Numbers on the right refer to the last amino acid in each corresponding line. Strictly conserved and conserved residues are shaded in black and gray, respectively. The identities and query cover between the SIR2 sequence of *E. tenella* Shanghai strain and the other species are listed at the end. Multiple alignments were made using ClustalX [51] and shaded using Boxshade 3.21 (http://www.ch.embnet.org/software/BOX_form.html). GenBank accession numbers for the *Eimeria* species are as follows: E*. maxima* Weybridge strain SIR2: CDJ59951; *E. acervulina* (Ea) Houghton strain SIR2: CDI78174; *E. praecox* (Ep) Houghton strain SIR2: CDI74590; *E. tenella* (Et) Shanghai strain SIR2A: KU871068; *E. tenella* Houghton strain SIR2: CDJ42555; *E. necatrix* (En) Houghton strain SIR2: CDJ65355. *Red boxes* indicate the conserved catalytic domain of a canonical sirtuin. *Black boxes* indicate the highly conserved regions within parasitic protozoa. Asterisks indicate the perfectly conserved residues within Apicomplexa [18]

Sequence analysis of the EtSIR2A open reading frame identified a polypeptide of 302 amino acid residues with a predicted molecular mass of 33.37 kDa. In a search against the GenBank database, the protein showed 100% (query cover 100%) identities with the putative NAD^+^-dependent deacetylase of *E. tenella* (Fig. 1). No signal peptide or transmembrane helix was present. The predicted motif structure indicated that EtSIR2A contained a SIR2 domain (residues 36–213) and contained GXGXS, FR, TQNXDXL, HG and GXS motifs responsible for NAD-binding and acetyl-lysine binding, as found in most eukaryotic sirtuins. In addition, EtSIR2A included a conserved C4 zinc-finger domain (Cys140, Cys143, Cys167 and Cys169) essential for zinc binding, and the 20 residues perfectly conserved within apicomplexan parasites.

### Expression and purification of recombinant EtSIR2A protein

rEtSIR2A was expressed in *E. coli* after induction with 1 mM IPTG at 37 °C for 6 h. rEtSIR2A was equally expressed in the sediment and supernatant. Purified protein was obtained from the supernatant using a His Bind Purification kit. The molecular mass of rEtSIR2A was about 37 kD (containing the His-tag), corresponding to the predicted molecular mass (Additional file 1: Figure S1).

### Transcriptional levels of EtSIR2A at different developmental stages of *E. tenella*

**Fig. 2:**
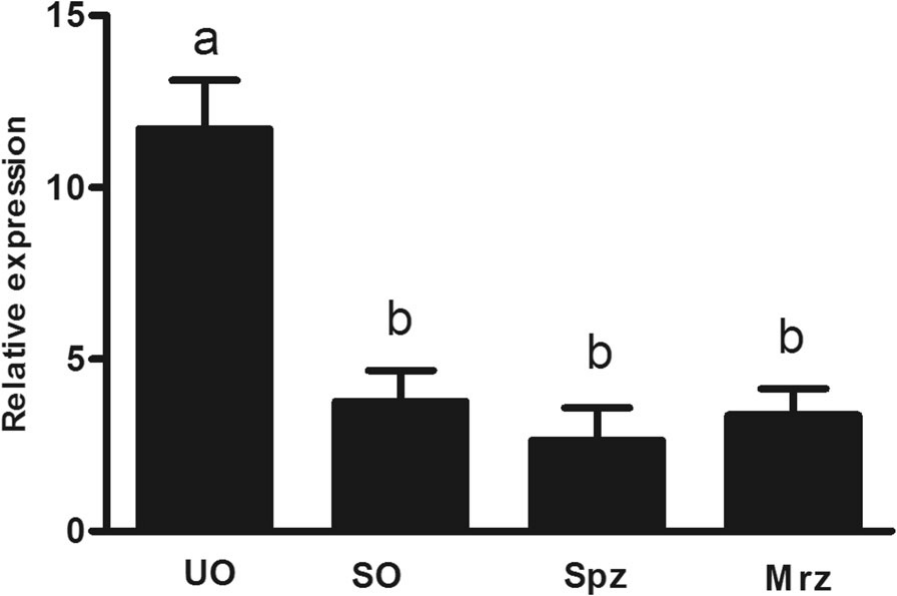
qPCR analysis of EtSIR2A mRNA transcript levels in different developmental stages of *E. tenella*. Bars not sharing the same letters indicate significantly different expression levels (*P* < 0.05). *Abbreviations*: UO, unsporulated oocysts; SO, sporulated oocysts; Spz, sporozoites; Mrz, second-generation merozoites

The mRNA levels of EtSIR2A at different developmental stages (unsporulated oocysts, sporulated oocysts, sporozoites and second-generation merozoites) were determined by qPCR. The transcriptional level was highest in unsporulated oocysts (*F*_(3,8)_ = 50.02, *P* < 0.0001), with no differences among sporulated oocysts, sporozoites and second-generation merozoites (Fig. 2) (*F*_(2,6)_ = 1.282, *P* = 0.344).

### Protein levels of EtSIR2A at different developmental stages of *E. tenella*

**Fig. 3:**
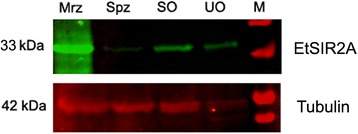
Immunoblot of native protein of EtSIR2A in different developmental stages of *E. tenella. Abbreviations*: UO, unsporulated oocysts; Spz, sporozoites; SO sporulated oocysts; Mrz, second-generation merozoites

We determined if EtSIR2A protein expression differed among different developmental stages of *E. tenella* by western blot analysis of extracts from unsporulated oocysts, sporulated oocysts, sporozoites and second-generation merozoites. EtSIR2A was detected in all extracts using a specific serum produced against rEtSIR2A. However, the protein expression levels differed significantly, with highest expression in second-generation merozoites, and weakest expression in sporozoites (Fig. 3).

### Immunofluorescence localization of EtSIR2A during first schizogony

**Fig. 4:**
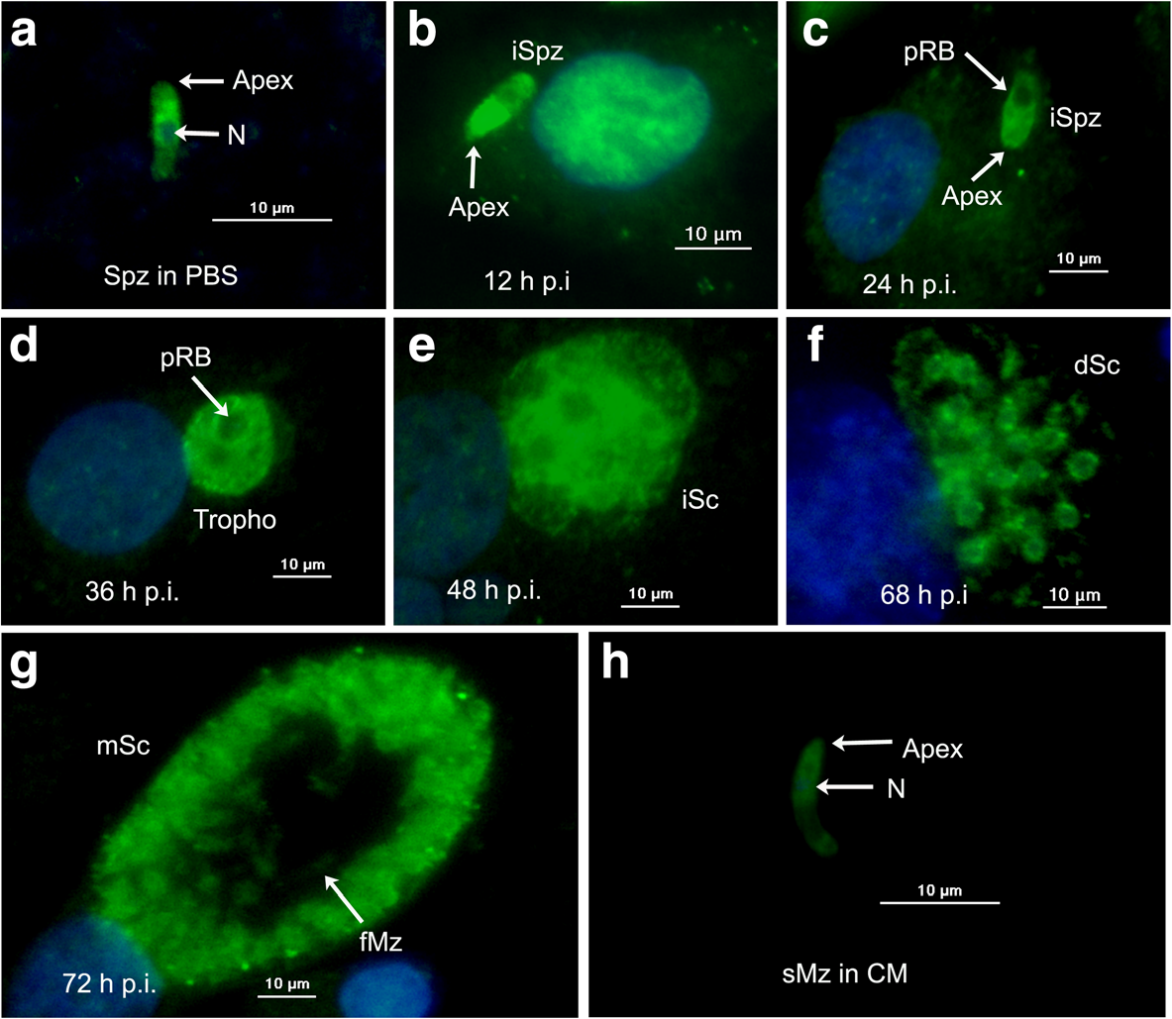
Localization of EtSIR2A in infected DF-1 cells by indirect immunofluorescence. Details of parasites immunostained with anti-rEtSIR2A antibodies visualized with fluorescein isothiocyanate (*green*) and counterstained with DAPI (*blue*). **a** Sporozoites (Spz) incubated in PBS. Infected DF-1 cells were collected at different times post-infection (p.i.). **b** 12 h p.i., intracellular sporozoites (iSpz); **c** 24 h p.i., intracellular sporozoites (iSpz); **d** 36 h p.i., trophozoites (Tropho); **e** 48 h p.i., immature first-generation schizonts (iSc); **f** 68 h p.i., developing first-generation schizonts (dSc); **g** 72 h p.i., mature first-generation schizonts (mSc); **h** 112 h p.i., second-generation merozoites (sMz) purified from caeca. *Abbreviations*: N: nucleus; pRB, posterior refractile body; fMz, first generation merozoites

The localization of EtSIR2A in sporozoites, second-generation merozoites, and during first schizogony was investigated by an indirect immunofluorescence assay using anti-rEtSIR2A as a probe. EtSIR2A protein was mainly located in the cytoplasm of free sporozoites (Fig. 4a) and intracellular ones (Fig. 4b, c). Labeled EtSIR2A was distributed throughout the whole cytosol in trophozoites, except in the refractile bodies (Fig. 4d). EtSIR2A protein was strongly expressed in immature and mature first-generation schizonts (Fig. 4e-g), and was mainly concentrated in cytoplasm in second-generation merozoites after culture in CM (Fig. 4h).

### Characterization of constructed plasmids in vitro

Expression of encoded EtSIR2A protein by the plasmids was confirmed by IFA and western blotting after transfection of DF-1 cells for 48 h. The intensive fluorescence detected in DF-1 cells transfected with pCAGGS-EtSIR2A indicated successful expression of the EtSIR2A protein (Additional file 2: Figure S2). Western blotting of lysates of DF-1 cells transfected with the EtSIR2A-encoding plasmids produced a band with the expected size of 37 kDa, while no band was present in the cells transfected with pCAGGS (Additional file 3: Figure S3). These results demonstrated that pCAGGS-EtSIR2A protein could be expressed in vitro.

### Protective efficacy of pCAGGS-EtSIR2A vaccination against *E. tenella* in chickens

The protective efficacy of pCAGGS-EtSIR2A following virulent *E. tenella* infection were determined. As shown in Table 1, the body-weight gain, caecal-lesion score, and oocyst output among the different groups were differed significantly (*F*_(3,96)_ = 53.394, *P* < 0.0001; *F*_(3,96)_ = 48.477, *P* < 0.0001; and *F*_(3,8)_ = 626.943, *P* < 0.0001, respectively). After challenge, chickens vaccinated with pCAGGS-EtSIR2A gained significantly more body weight, and had significantly fewer caecal lesions (Additional file 4: Figure S4) and oocysts compared with chickens vaccinated with pCAGGS or TE-challenged controls. Oocyst shedding in the pCAGGS group was significantly lower than in the TE-challenged control groups, but there were no significant differences in terms of body weight and caecal lesions.

**Table 1:** Protective effects of pCAGGS-EtSIR2A against experimental *Eimeria tenella* infection in chickens

Group	Average body weight gain (g)	Reduced rate of weight gain (%)	Oocyst shedding per bird (10^7^)	Oocyst decrease (%)	Lesion scores
pCAGGS-EtSIR2A	97.42 ± 6.56^a^	23.62	1.93 ± 0.15^b^	62.96	1.09 ± 0.67^b^
pCAGGS	75.61 ± 11.79^b^	40.72	4.02 ± 0.26^c^	22.84	2.20 ± 0.42^c^
TE challenged	64.93 ± 18.53^b^	49.09	5.21 ± 0.38^d^	0	2.30 ± 0.68^c^
TE unchallenged	127.54 ± 15.55^a^	0	0.0 0.00^a^	100	0.0 0.00^a^

Values are expressed as mean ± standard deviation (SD). Means in the same column with different letters were significantly different between treatment groups (*P* < 0.05)

## Discussion

Sirtuin family members share a catalytic domain that allows them to function as NAD + -dependent protein deacetylases [18]. The sirtuin core domain includes several short motifs of conserved amino acids, including GAGISXXXGIPXXR, PXXXH, TQNID, HG, two sets of CXXC that may be a zinc-finger domain [33], FGE, GTS and (I/V)N [7]. Sirtuin proteins have been classified into five different classes (I, II, III, IV and U), depending on the presence of specific conserved motifs in their core domain. In class III sirtuins, the GAGISXXXGIPXXR and PXXXH motifs are usually GAGISAESGIPTFR and PNXXH, respectively. The presence of GIPT within the former motif is indicative of a class III sequence [7]. Most apicomplexans possess two sirtuins, SIR2A and SIR2B. SIR2A sirtuin domains can be assigned to class III or class U, and SIR2B domains to class IV. Twenty residues were found to be perfectly conserved within apicomplexan parasite sirtuins [18]. The *E. tenella* genome may encode two SIR2-like proteins, though the current genome assembly status only allows the confident assignment of one of them to SIR2A type (ETH 00033350), while the other resembles a SIR2B type, based on the small portion of available sequence (ETH 00041870) [18].

In the present work, we cloned and characterized the SIR2A gene from *E. tenella* Shanghai strain. The obtained sequence showed 100% identity with the published NAD^+^-dependent deacetylase (SIR2) sequence from *E. tenella*. The putative NAD^+^-dependent deacetylase genes of *E. maxima*, *E. acervulina, E. praecox* and *E. necatrix* have also been deposited in the GenBank database, and the primary amino acid sequences of these five *Eimeria* species showed 76–99% homology, demonstrating that SIR2A is conserved among different *Eimeria* species. Sequence analysis showed that EtSIR2A was broadly conserved at the most invariant motifs of class III sirtuin, with only two substitutions: GAG*I*SAESGIPTFR/GAG*V*SAESGIPTFR and TQN*I*D/TQN*V*D. EtSIR2A also contained the 20 residues perfectly conserved within apicomplexan parasites. The most common sirtuin inhibitor nicotinamide was also an effective inhibitor of EtSIR2A [19]. Phylogenetic analysis showed that EtSIR2A was closer to those of *Toxoplasma* and *Neospora* than *Plasmodium* [19], which agrees with *Eimeria* spp. having closer evolutionary relationships with *T. gondii* and *N. caninum* than with other apicomplexan species [34]. EtSIR2A showed low identity (25%) with chicken SIR, indicating different kinetic and structural properties, and suggesting that EtSIR2A may represent a novel anti-coccidial therapeutic target [19].

The life-cycle of *E. tenella* involves endogenous (schizogamy and gametogony) and exogenous (sporogony) developmental stages [35]. qPCR and western blotting demonstrated that EtSIR2A mRNA and protein were expressed in unsporulated oocysts, sporulated oocysts, sporozoites and second-generation merozoites in *E. tenella*, suggesting that it performs an important cellular function. However, the expression levels were significantly different; the highest transcript levels were detected in unsporulated oocysts, with lower levels in sporulated oocysts, sporozoites and second-generation merozoites, while the highest protein levels occurred in second-generation merozoites, with the lowest levels in sporozoites. The fact that the transcript and protein expression levels of EtSIR2A were not in accordance suggests that EtSIR2A protein expression may be controlled by post-transcriptional regulation of translation. Overall, these results indicate that the gene and protein expression levels of EtSIR2A are developmentally regulated. Similar results were obtained for sirtuins from other parasites. Five sirtuins (SmSirt) are encoded in the *Schistosoma mansoni* (Sm) genome, all five of which are expressed throughout the parasite’s life-cycle (miracidia, sporocysts, cercariae, schistosomula and adult worms), but with distinct patterns of expression [36]. *Trypanosoma cruzi* (Tc) has two sirtuins, TcSir2rp1 and TcSir2rp3. TcSir2rp1 was shown to be highly expressed in replicative parasite forms (epimastigotes and amastigotes) and less in metacyclic trypomastigotes and trypomastigotes derived from infected mammalian cells, while TcSir2rp3 was largely expressed in epimastigotes and cell-derived trypomastigotes, and much less in intracellular amastigotes and metacyclic trypomastigotes [37].

SIR2 proteins present a broad spectrum of intracellular localizations. Some occur in the nucleus, while others seem to be mainly cytoplasmic, such as yeast Hst2p [38], *Leishmania* SIR2 proteins [39] and human SIRT2 [40, 41]. A mitochondrial localization has also been described for human SIRT3 [42]. Different sirtuins thus function differently in line with their different intracellular localizations. Immunolocalization studies in *E. tenella* showed that EtSIR2A protein was mainly located in the cytoplasm of free sporozoites incubated in PBS and intracellular sporozoites. EtSIR2A protein was strongly expressed during first schizogony and was mainly located in cytoplasm of second-generation merozoites after culture in CM. These results suggest that EtSIR2A may play an important role in parasite cell survival, but further studies are needed to confirm this.

Previous studies showed that SIR2 promoted parasite survival under various conditions [11–16], and may thus serve as a therapeutic target for developing novel anti-parasitic drugs [10, 17]. *Leishmania* sirtuins from *L. major* and *L. infantum* have demonstrated roles in parasite growth in vitro and in vivo. Involvement of the protein in parasite virulence and survival [11, 12] suggests that it may be exploited as a novel vaccine target against leishmaniasis [43]. Recombinant SIR2 protein was reported to be capable of inducing the activation and differentiation of B cells, thus producing specific antibodies [44], and demonstrated a protective function in BALB/c mice by reducing the parasite load after *L. infantum* infection [45]. In the present study, we generated a recombinant chimeric subunit vaccine, consisting of EtSIR2A and a eukaryotic expression vector, and evaluated its efficacy against *E. tenella* infection in chickens.

The eukaryotic expression vector used in this study was pCAGGS, which contains the cytomegalovirus enhancer and chicken *β-actin* promoter sequence in the upstream of its multiple cloning site. pCAGGS vectors have been widely used in the development of DNA vaccines, for example in relation to reticuloendotheliosis virus [46], infectious bursal disease virus [47], avian influenza virus [48], swine influenza virus [49] and duck tembusu virus [50]. We confirmed the expression of EtSIR2A protein by in vitro methods before carrying out in vivo experiments. Intensive fluorescence in DF-1 cells transfected with pCAGGS-EtSIR2A and the presence of a 37-kDa band in the cell lysates indicated that the recombinant plasmid pCAGGS-EtSIR2A was successfully constructed and expressed in eukaryotic cells. The results of the challenge experiments showed that chickens treated with the DNA vaccine gained significantly more weight, and had significantly fewer caecal lesions and oocysts, compared with infected chickens treated with the control vaccine. The results of this study thus suggest that EtSIR2A might be an effective candidate antigen for the development of a new vaccine against *E. tenella* in chickens.

## Conclusions

The present study showed that the mRNA and protein levels of EtSIR2A were developmentally regulated at different developmental stages of *E. tenella.* EtSIR2A was located mainly in the cytoplasm of free sporozoites and intracellular sporozoites. EtSIR2A protein was strongly expressed during first schizogony and was mainly located in cytoplasm in second-generation merozoites. The recombinant plasmid pCAGGS-EtSIR2A induced a partial protective immunity the immunized chickens. Our results suggested that EtSIR2A may play an important role in parasite cell survival and may be an effective candidate for the development of new vaccines against *E. tenella* infection in chickens.

## Additional files

Additional file 1: Figure S1. Expression analysis of EtSIR2A in *E. coli* BL21 (DE3) using SDS-PAGE. Lane 1, protein marker; Lanes 2, 3 and 4, induced with IPTG at 6, 4, 2 h, respectively; Lane 5, negative control (not induced with IPTG). (PDF 157 kb)
Additional file 2: Figure S2. Indirect immunofluorescence assay of encoded EtSIR2A protein in transfected DF-1 cells. **a** pCAGGS-EtSIR2A-transfected DF-1 cells. **b** pCAGGS-transfected DF-1 cells. (PDF 166 kb)
Additional file 3: Figure S3. Western blot analysis of EtSIR2A protein expressed from pCAGGS-EtSIR2A in transfected DF-1 cells. Lane 1, cell lysates of pCAGGS-EtSIR2A-transfected DF-1 cells; Lane 2, cell lysates of pCAGGS-transfected DF-1 cells. (PDF 91 kb)
Additional file 4: Figure S4. Caecal lesion of pCAGGS-EtSIR2A vaccinated chicken against experimental *Eimeria tenella* infection. **a** pCAGGS-EtSIR2A vaccinated and challenged group. **b** pCAGGS vaccinated and challenged group. **c** TE vaccinated and challenged group. **d** TE vaccinated and unchallenged group. (PDF 1047 kb)

## Abbreviations

CM: Complete medium
DAPI: 4, 6-diamidino-2-phenylindole
DMEM: Dulbecco’s modified Eagle’s medium
Et: *Eimeria tenella*
FBS: Fetal bovine serum
IFA: Immunofluorescence assay
IPTG: Isopropyl-β-D-thiogalactopyranoside
PBS: Phosphate-buffered saline
PBST: PBS containing 0.1% Tween 20
PCR: Polymerase chain reaction
qPCR: Quantitative real-time PCR
SDS-PAGE: Sodium dodecyl sulfate–polyacrylamide gel electrophoresis
SIR2: Silent information regulator 2
Sm: *Schistosoma mansoni*
Tc: *Trypanosoma cruzi*
