# Supplementary material for: Molecular characterization and protective efficacy of silent information regulator 2A from Eimeria tenella
Source: Parasit Vectors. 2016 Nov 25;9:602. doi: 10.1186/s13071-016-1871-0 (PMC5123391; doi:10.1186/s13071-016-1871-0)
Supplement: Additional file 1: Figure S1. — Expression analysis of EtSIR2A in E. coli BL21 (DE3) using SDS-PAGE. Lane 1, protein marker; Lanes 2, 3 and 4, induced with IPTG at 6, 4, 2 h, respectively; Lane 5, negative control (not induced with IPTG). (PDF 157 kb) [file 13071_2016_1871_MOESM1_ESM.pdf]

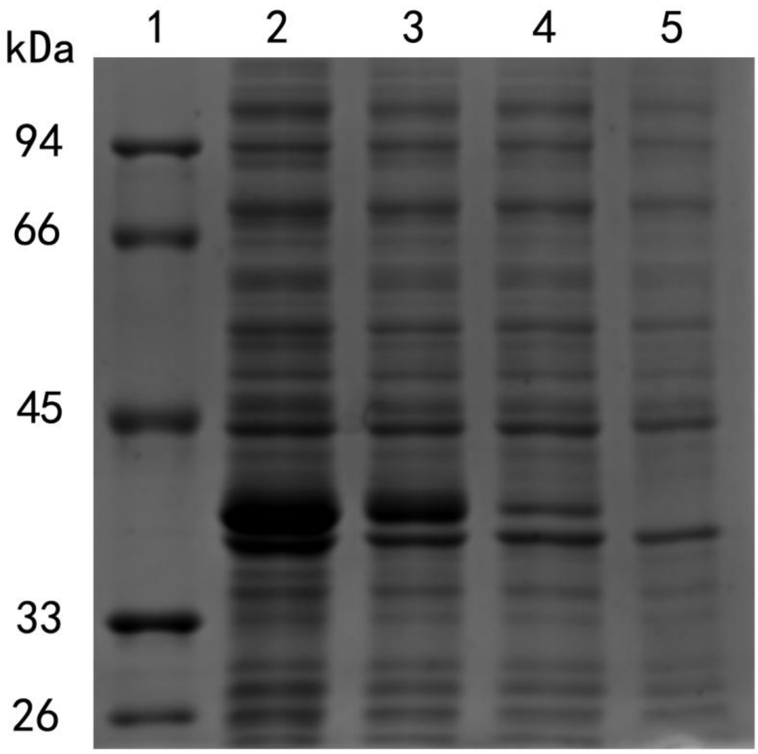

Figure S1. Expression analysis of EtSIR2A in *E. coli* BL21 (DE3) using SDS–PAGE. Lane 1, protein marker; Lanes 2, 3 and 4, induced with IPTG at 6, 4, 2 h, respectively; Lane 5, negative control (not induced with IPTG).
