# Supplementary material for: Molecular characterization and protective efficacy of silent information regulator 2A from Eimeria tenella
Source: Parasit Vectors. 2016 Nov 25;9:602. doi: 10.1186/s13071-016-1871-0 (PMC5123391; doi:10.1186/s13071-016-1871-0)
Supplement: Additional file 2: Figure S2. — Indirect immunofluorescence assay of encoded EtSIR2A protein in transfected DF-1 cells. a pCAGGS-EtSIR2A-transfected DF-1 cells. b pCAGGS-transfected DF-1 cells. (PDF 166 kb) [file 13071_2016_1871_MOESM2_ESM.pdf]

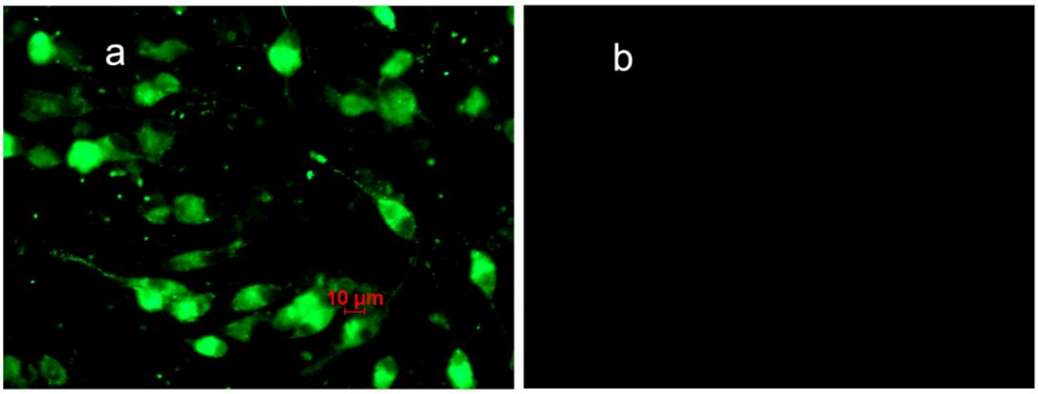

Figure S2. Indirect immunofluorescence assay of encoded EtSIR2A protein in transfected DF-1 cells. **a** pCAGGS-EtSIR2A-transfected DF-1 cells. **b** pCAGGS-transfected DF-1 cells.
