# Supplementary material for: Molecular characterization and protective efficacy of silent information regulator 2A from Eimeria tenella
Source: Parasit Vectors. 2016 Nov 25;9:602. doi: 10.1186/s13071-016-1871-0 (PMC5123391; doi:10.1186/s13071-016-1871-0)
Supplement: Additional file 3: Figure S3. — Western blot analysis of EtSIR2A protein expressed from pCAGGS-EtSIR2A in transfected DF-1 cells. Lane 1, cell lysates of pCAGGS-EtSIR2A-transfected DF-1 cells; Lane 2, cell lysates of pCAGGS-transfected DF-1 cells. (PDF 91 kb) [file 13071_2016_1871_MOESM3_ESM.pdf]

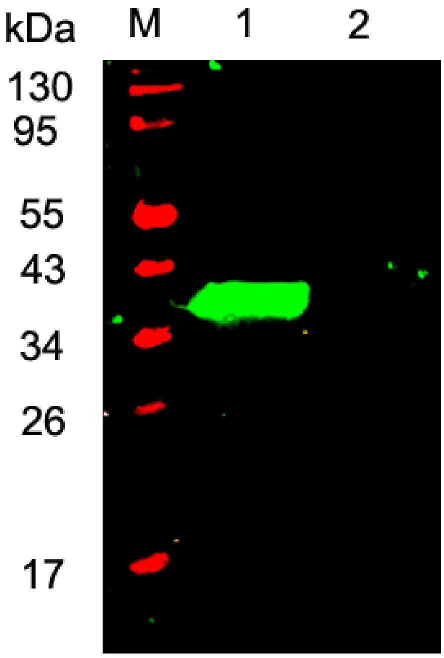

Figure S3. Western blot analysis of EtSIR2A protein expressed from pCAGGS-EtSIR2A in transfected DF-1 cells. Lane 1, cell lysates of pCAGGS-EtSIR2A -transfected DF-1 cells; Lane 2, cell lysates of pCAGGS- transfected DF-1 cells.
