# Supplementary material for: Molecular characterization and protective efficacy of silent information regulator 2A from Eimeria tenella
Source: Parasit Vectors. 2016 Nov 25;9:602. doi: 10.1186/s13071-016-1871-0 (PMC5123391; doi:10.1186/s13071-016-1871-0)
Supplement: Additional file 4: Figure S4. — Caecal lesion of pCAGGS-EtSIR2A vaccinated chicken against experimental Eimeria tenella infection. a pCAGGS-EtSIR2A vaccinated and challenged group. b pCAGGS vaccinated and challenged group. c TE vaccinated and challenged group. d TE vaccinated and unchallenged group. (PDF 1047 kb) [file 13071_2016_1871_MOESM4_ESM.pdf]

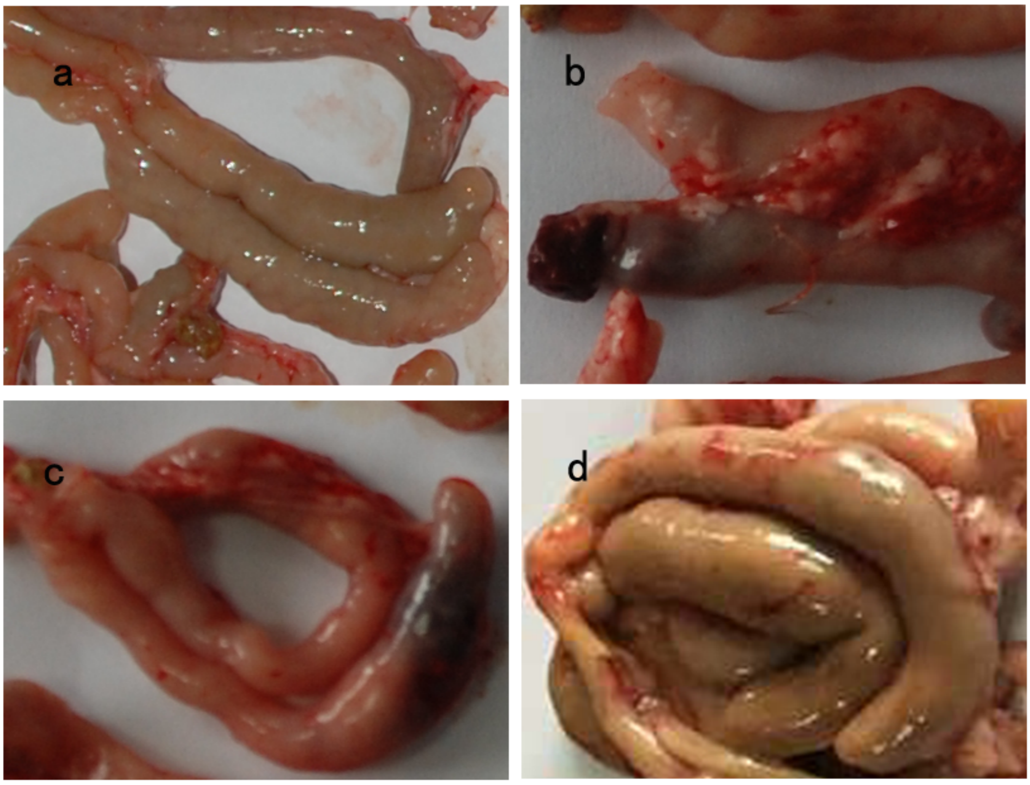


**Figure S4.** Caecal lesion of pCAGGS-EtSIR2A vaccinated chicken against experimental *Eimeria tenella* infection. **a** pCAGGS-EtSIR2A vaccinated and challenged group. **b** pCAGGS vaccinated and challenged group. **c** TE vaccinated and challenged group. **d** TE vaccinated and unchallenged group.
